# The Role of the Morphological Characterization of Multilayer Hydrophobized Ceramic Membranes on the Prediction of Sweeping Gas Membrane Distillation Performances

**DOI:** 10.3390/membranes12100939

**Published:** 2022-09-27

**Authors:** Mohamed K. Fawzy, Felipe Varela-Corredor, Cristiana Boi, Serena Bandini

**Affiliations:** Department of Civil, Chemical, Environmental and Materials Engineering, DICAM, Alma Mater Studiorum-University of Bologna, Via Terracini 28, 40131 Bologna, Italy

**Keywords:** hydrophobic ceramic membranes, multilayer membrane, membrane distillation, morphological parameters, modeling

## Abstract

This paper shows which morphological characterization method is most appropriate to simulating membrane performance in sweeping gas membrane distillation in the case of multilayer hydrophobized ceramic membranes. As a case study, capillary four-layer hydrophobic carbon-based titania membranes arranged in bundles in a shell-and-tube configuration were tested with NaCl-water solutions using air as sweeping gas, operating at temperatures from 40 to 110 °C and at pressures up to 5.3 bar. Contrary to what is generally performed for polymeric membranes and also suggested by other authors for ceramic membranes, the mass transfer across the membrane should be simulated using the corresponding values of the mean pore diameter and the porosity-tortuosity ratio of each layer and measured by the layer-by-layer (LBL) method. Comparison of the modeling results with experimental data highlights that the use of parameters averaged over the entire membrane leads to an overestimation by a factor of two to eight of the modeled fluxes, with respect to the experimental values. In contrast, the agreement between the modeled fluxes and the experimental values is very interesting when the LBL parameters are used, with a discrepancy on the order of +/−30%. Finally, the model has been used to investigate the role of operative parameters on process performances. Process efficiency should be the optimal balance between the concomitant effects of temperature and velocity of the liquid phase and pressure and velocity of the gas phase.

## 1. Introduction

The application of membrane distillation (MD) in water desalination has attracted many researchers because of its advantages over conventional methods in terms of high salt rejection and possible energy savings if a low-grade waste heat source is available [1,2].

Sweeping gas membrane distillation (SGMD) is one of the typical configurations of the MD technique, which is performed with hydrophobic membranes, as is the case with all membrane distillation processes [2,3,4,5]. An aqueous liquid stream is immobilized at the inlet of the membrane pores; the liquid vaporizes at the liquid/membrane interface, and the vapors diffuse through a stagnant film of gas contained in the membrane pores and into the gas stream flowing in the permeate side. In the case of non-volatile solutes, such as salts, only water vapor permeates the membrane, and a desalination process can be developed up to high values of salt concentration and with high levels of permeate purity. 

SGMD can be considered a hybrid of thermal distillation and membrane separation technology. It is a thermally driven process, in that heat must be provided for vaporization, but it has aspects typical of membrane technology, since diffusive mass transfer occurs across the membrane pores under a partial pressure difference driving force [2,3,4,6].

A key requirement for performing SGMD is the hydrophobic character of the material, which is essential to prevent liquid intrusion into the membrane pores, provided that the transmembrane pressure difference is less than a certain value, known as minimum liquid entry pressure *(LEP_min_*) [2,3,4]. Polymeric membranes, such as polypropylene, polytetrafluoroethylene, or polyvinylidene difluoride, and its modifications are typically used in MD operations because they have an *LEP_min_* at room temperature higher than 2–3 bar [3,7,8,9,10]. 

Compared with polymeric materials, ceramic membranes (alumina, zirconia, titania, and silica, or a combination thereof) hydrophobized with fluoroalkylsilanes and/or by carbonization techniques have been proposed as alternatives due to their higher mechanical and thermal stability associated with increased chemical stability and lifetime [11,12,13,14,15,16,17,18,19]

Since ceramic membranes are typically asymmetric, formed by the deposition of several layers, when developing an SGMD process with ceramic modules, there are essentially two types of problems: membrane characterization and process simulation. 

Membrane characterization must evaluate the hydrophobic character of the material and provide the morphological parameters of the membrane.

Although ceramics can provide the high thermal stability required for flux enhancement, operation at relatively high feed temperatures may prove counterproductive, as *LEP_min_* decreases with increasing temperature. A critical wetting temperature in the range from 130 to 135 °C was found for FAS-grafted carbon-based membrane titania [20,21], indicating that maximum operating temperature values should not exceed 100–120 °C, values at which *LEP_min_* drops below 1 bar. Similar values have been obtained for alumina membrane grafted with FAS [22]. 

The morphological parameters to be considered are the mean pore diameter, the thickness and the porosity-tortuosity ratio of each membrane layer. The thickness is generally obtained by SEM imaging, while the pore diameter and porosity-tortuosity ratio should be obtained by gas permeation tests, processed according to the “layer-by-layer” method, as discussed by Fawzy et al. [23]. In the same work, the authors showed that this method is more appropriate than the traditional method, typically applied for polymeric membranes [4,24] and re-proposed by Koonaphapdeelert and Li [17] for hydrophobized ceramic membranes, in which the average properties over the entire membrane were evaluated. 

A reliable investigation of the effect of membrane properties, operating conditions, and module configuration on the process requires the coupling of robust experimental and modeling studies. 

Regarding process simulation, basic modeling of MD processes has been known since the last century [4]; however, more recently, Karanikola et al. [25] used a different approach to the modeling and experimental studies for SGMD using PVDF hollow fibers. Fractal theory was adopted to estimate the mass transfer resistance in the shell-side (permeate side) to account for the random distribution of fibers in the shell. CFD simulation studies were conducted by Elsheniti et al. [26] to investigate the effect of turbulators in the permeate side on flux enhancement during SGMD, with a 39% increase in flux at a feed temperature of 70°C. 

For non-homogeneous multilayer membranes, such as hydrophobized ceramic membranes, modeling transmembrane flux in SGMD operation should require knowledge of morphological parameters to calculate mass and heat transfer across the membrane. 

The main objective of this paper is to critically discuss the accuracy of the two different procedures for characterizing morphological parameters. A clear conclusion is reached on which method is most appropriate by testing tubular modules containing hydrophobized carbon-based titania membranes that represent the originality of this work. The discussion is carried out by simulating the performance of the modules in the SGMD of NaCl-water solutions according to the same transport equations, using different morphological parameters that were obtained, for the same modules, in a previous characterization with the “layer-by-layer” method and with the “average-membrane-morphology” method [23]. The simulations are compared with the experimental results obtained in the SGMD of NaCl-water solutions with air as sweeping gas, operating at temperatures from 40 to 110 °C, and at pressures up to 5.3 bar. 

Finally, the developed SGMD model was used to study the effect of some relevant operating conditions on the water flux achievable with modules of the same type.

## 2. Materials and Methods

### 2.1. Membranes and Modules

**Figure 1:**
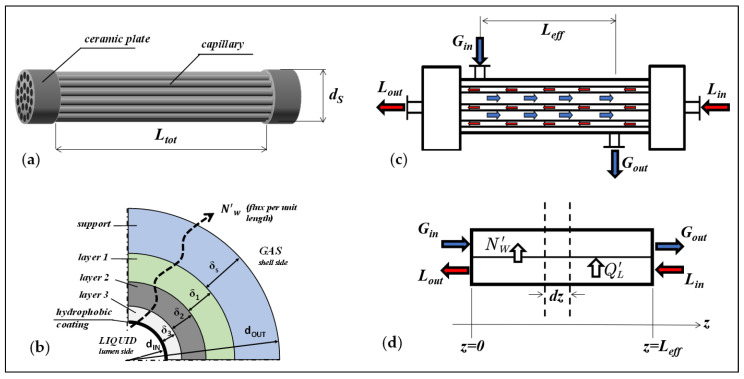
Scheme of membranes and modules: (**a**) Capillary bundle; (**b**) Cross-section of the multilayer membrane of a capillary; (**c**) Bundle-housing arrangement in counter-current flow pattern; (**d**) System coordinates for the equations used in the plug-flow (parallel flow) model.

The experimental and modeling studies of SGMD in the present work were carried out on five capillary bundles, made of hydrophobized carbon-based titania membranes, manufactured by the Fraunhofer Institute for Ceramic Technologies and Systems (IKTS, Hermsdorf, Germany). The schematic of the membranes and modules is shown in Figure 1.

The membranes are composed of four layers (Figure 1b) with different morphological properties. The layers are arranged according to their proximity to the liquid feed: “layer 3”, “layer 2”, “layer 1”, and “support”, as it can be observed in the SEM images reported in [27]. The fabrication of the capillary bundles was described by the manufacturer in [27,28,29], the titania membranes were coated with carbon by deposition and pyrolysis of a polymeric precursor according to the patent [13], and then grafted on the surface- with a fluoroalchylsilane (FAS, tridecafluoro-1,1,2,2-tetra-hydro-octyl-trichloro-oxysilane) according to the patent [30] to give the membranes the hydrophobic character; details about the hydrophobization procedures are also provided in [31,32].

The geometrical parameters of the bundles are listed in Table 1. They include the inner diameter (*d_IN_*) and outer diameter (*d_OUT_*) of the fiber, number of fibers (*N_f_*), the inner surface area (*A_IN_*), the total fiber length (*L_tot_*), and the inner shell diameter (*d_S_*), as depicted in Figure 1a. 

The capillary bundles are arranged in a shell-and-tube configuration un-baffled housing (Figure 1c) and were used in a countercurrent flow pattern, in which liquid flows in the lumen side and gas in the shell-side. The distance between the inlet and outlet nozzles of the shell represents the effective mass transfer length of the module (*L_eff_*). 

The applicability of the bundles for membrane distillation operations is confirmed by the minimum liquid entry pressure (*LEP_min_*) values obtained with pure water according to the “flooding curve” method introduced by Varela-Corredor et al. [20,21] (Table 1). In agreement with the results obtained by Varela-Corredor et al. [21], in which the critical wetting temperature for that material was measured in the range of 130 to 135 °C, all the bundles used in the present work show the required hydrophobic character when employed at pressures below *LEP_min_*. 

Morphological characterization of the hydrophobized bundles was reported in a previous paper of our group [23], in which the authors complemented, by air permeation tests, the “layer-by-layer” (LBL) characterization of the capillaries, previously presented by M.Weyd et al. [27]. The pore size and the porosity-tortuosity ratio of “layer 3” were estimated by the elaboration of the air permeation data, according to the Dusty Gas Model. Results are summarized in Table 2.

In the same paper, the authors also show the results of the mean morphological parameters obtained by elaborating the same air permeance data, according to the Dusty Gas Model, on the whole membrane, following the typical protocol in which the membrane is schematized as consisting of a single hypothetical layer with average morphological properties [4,17,24]. The mean porosity-tortuosity ratio (*(ε/τ)_m_*) and the mean pore diameter (*dp_m_*) calculated according to this method, abbreviated in the following as the “average-membrane-morphology” (AMM) method, are listed in Table 3 for each bundle.

### 2.2. Experimental Set-Up and Procedures

**Figure 2:**
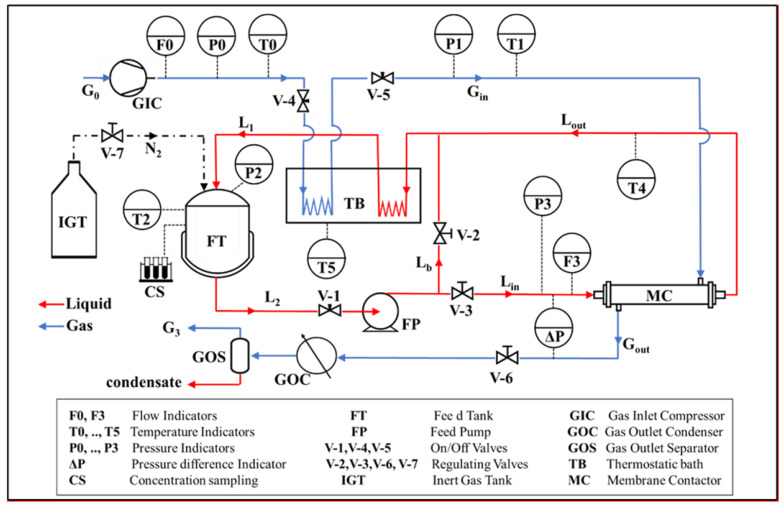
Diagram of the SGMD experimental set-up.

The bench-scale SGMD configuration depicted in Figure 2 was used for experimentation. An aqueous NaCl solution was loaded into the feed tank (FT: 5 L maximum capacity) and continuously recirculated to the lumen-side of the bundle (MC). The sweeping gas stream (dry air) was fed to the bundle in single-pass mode; a condenser (GOC) in the gas phase downstream of the module was used to recover the permeate as a liquid phase and to check for salt. Throughout the experimentation, the absence of NaCl in the condensate was verified, documenting complete salt rejection by the membranes.

All elements employed in the SGMD pilot plant were constructed of AISI316L and were designed to withstand up to 10 bar and 150 °C. Liquid and gas streams temperatures were controlled by a thermostatic silicon oil bath. The pressure (P0) and flow rate (F0) of the inlet air were controlled; the gas stream was typically fed at room temperature (T0). With regards to the liquid stream, the temperature (T2) in the reservoir and the liquid flow rate (F3) at the module inlet section were controlled, as well as the pressure inside the feed tank (P2) by pressurized nitrogen.

The differential manometer (ΔP) was used for continuous monitoring of the maximum transmembrane pressure difference, which, in the countercurrent flow pattern, is the difference between the liquid stream inlet section and the gas stream outlet section. The main purpose of this measurement was to ensure that it did not exceed *LEP_min_* at the corresponding liquid-side temperature to avoid membrane wetting.

The experiments were conducted with the bundles (Table 1) at different conditions for liquid and gas streams. The summary of operating conditions is given in Table 4. Typically, the inlet liquid temperature and pressure ranged from 50 to 110 °C and from 2.3 to 5.2 bar, respectively. Whereas, the inlet gas temperature and pressure ranged from 40 to 70 °C and from 1.9 to 5.3 bar, respectively. It is important to note that at the maximum liquid temperature of 110 °C, the ΔP value approached 0.33 bar, which is rather lower than the corresponding *LEP_min_* value of 0.9 bar, as reported in [21].

The experiments were performed with a nominal NaCl concentration of 20 g/kg. The salt concentration was measured by a conductivity meter. After the solution was loaded into the tank, the liquid was recirculated and the salt concentration in the tank was measured by taking samples at regular time intervals. At the same time, the temperature trend of the liquid and gas is measured as a function of time. The tests lasted between 360 and 450 min. This procedure allowed for a large data set, characterized by different temperature values and corresponding NaCl salinity (*S_NaCl_*), as shown in Table 4. 

For each test, the calculation of water flux through the membrane was performed by means of a salt mass balance on the liquid phase, based on the measurement of the salt concentration in the liquid tank on two subsequent samples; the liquid phase was considered as a perfectly mixed solution and a complete NaCl rejection was taken into account, as detected by the absence of salt in the GOC condensate.

The modules operated for 2–3 days each without showing wetting phenomena.

For two successive samples, the time at which the first sample was taken from the feed tank is indicated as (*t*_1_), and the time at which the second sample was taken is referred to as (*t*_2_). Consequently, the salinity of NaCl (*S_NaCl_*) is measured at time (*t*_2_), and the total mass in the liquid phase (*m_tot, liquid phase_(t*_2_)) can be evaluated, assuming total salt rejection, as shown in Equation (1), since the total mass of NaCl contained in the liquid side is known as the initial value. The experimental water flux (*J_w_*) through the bundle during the period (*t*_2_ − *t*_1_) can be finally calculated according to Equation (2), with reference to the inner surface.

(1) $$m_{tot,liquid\;phase}(t_{2})\times S_{NaCl}(t_{2})=m_{tot,liquid\;phase}(t_{1})\times S_{NaCl}(t_{1})=m_{NaCl,initial}$$

(2) $$J_{w}=\frac{m_{w,liquid\;phase}(t_{1})-m_{w,liquid\;phase}(t_{2})}{A_{IN}\times (t_{2}-t_{1})}=\frac{m_{tot,liquid\;phase}(t_{1})-m_{tot,liquid\;phase}(t_{2})}{A_{IN}\times (t_{2}-t_{1})}$$

## 3. SGMD of NaCl-Water Solutions across Capillary Bundles: Model Equations

In this section, we report the equations needed to simulate the performance of the modules to carry out the comparison between experimental data and simulations with membrane parameters obtained with the LBL and AMM methods, which will be reported in Section 4. The experimental data are obtained for SGMD of NaCl-water solutions.

The basic assumptions for the model are the following:

- Steady-state conditions;
- Total NaCl rejection: the membrane is a perfect barrier and thus only water permeates;
- Gas phase behaves as an ideal gas mixture;
- No heat loss in the module (well-insulated module);
- Parallel flow of liquid and gas streams within the module.

### 3.1. Local Model: Heat and Mass Transfer across The Membrane

**Figure 3:**
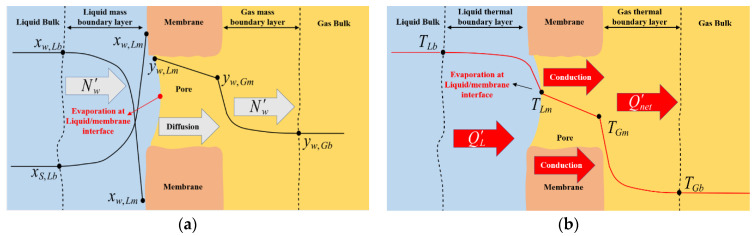
SGMD of water-salt solutions: expected (**a**) Composition profiles; (**b**) Temperature profiles across a generic membrane section.

With reference to a generic cross-section of the membrane, local model equations are developed taking into account the diagrams and notation shown in Figure 3. 

It is known that mass transport in SGMD is represented by a diffusive transport with a composition gradient as the driving force across the membrane [3,4]. In the case of SGMD of salt-water mixtures through a cylindrical membrane, the molar flux of water per unit length of a single capillary (*Nʹ_w_*) can be described as the combination of molecular and Knudsen diffusion through a stagnant gas (air) [23,33], represented by Equation (3), in which the mass transfer coefficient of the membrane (*k_w,m_*) is defined in a direct way.

(3) $$N_{w}^{\prime }=\frac{k_{w,m}\;P_{G}}{R_{g}T_{m}}\ln\left(\frac{1-y_{w,Gm}}{1-y_{w,Lm}}\right)\pi d_{lm,m}$$

In the case of multilayer ceramic membranes, the mass transfer coefficient of the membrane can be expressed according to several relationships that must consider the different method of morphological characterization.

Using the morphological parameters of each layer obtained from LBL characterization, as reported in Table 2, *k_w,m_* can be expressed by the relations reported in Equation (4):

(4) $$\begin{array}{l} \frac{1}{k_{w,m}\;d_{lm,m}}=\sum_{j=1}^{3,s}\frac{1}{k_{w,j}\;d_{lm,j}};k_{w,j}={\left(\frac{\varepsilon }{\tau }\right)}_{j}\frac{D_{Weq,j}}{\delta_{j}}\\ \mathrm{with}\\ \frac{1}{D_{Weq,j}}=\frac{1}{D_{WG}}+\frac{1}{D_{W,Kn,j}}\;;D_{W,Kn,j}=\frac{d_{p,j}}{3}\sqrt{\frac{8R_{g}T_{m}}{\pi M_{W}}} \end{array}$$

where “*j*” represents a single layer and/or the support, as indicated in Figure 1b; the equivalent diffusivity of water in layer *j* (*D_Weq,j_*) can be estimated from the Bosanquet equation [4,34] by using the Knudsen diffusivity of each layer *j* (*D_W,Kn,j_*) and the molecular diffusivity of water in air (*D_WG_*). The membrane temperature (*T_m_*) is calculated as the arithmetic mean of the temperatures at the two membrane interfaces (*T_Lm_*) and (*T_Gm_*).

Conversely, using the morphological parameters obtained from the AMM characterization given in Table 3, *k_w,m_* can be expressed by the relations reported in Equation (5), in which the average values of the pore diameter (*d_pm_*) and the porosity-tortuosity ratio ((*ε/τ*)_*m*_) of the membrane are used.

(5) $$\begin{array}{l} k_{w,m}={\left(\frac{\varepsilon }{\tau }\right)}_{m}\frac{D_{Weq,m}}{\delta_{m}};\delta_{m}=\delta_{s}+\delta_{1}+\delta_{2}+\delta_{3}\\ \mathrm{with}\\ \frac{1}{D_{Weq,m}}=\frac{1}{D_{WG}}+\frac{1}{D_{W,Kn,m}}\;;D_{W,Kn,m}=\frac{d_{pm}}{3}\sqrt{\frac{8R_{g}T_{m}}{\pi M_{W}}} \end{array}$$

The diffusive mass transfer across the membrane must be coupled with the mass transfer in the liquid phase boundary layer (Equation (6)) and the mass transfer in the gas permeate side boundary layer (Equation (7)), according to the premises of the film theory model:

(6) $$N_{w}^{\prime }=\frac{k_{S,L}\;\rho_{L}}{M_{L}}\ln\left(\frac{1-x_{w,Lm}}{1-x_{w,Lb}}\right)\pi d_{IN}$$

(7) $$N_{w}^{\prime }=\frac{k_{w,G}\;P_{G}}{R_{g}T_{G}}\ln\left(\frac{1-y_{w,Gb}}{1-y_{w,Gm}}\right)\pi d_{OUT}$$

in which *k_S,L_* and *k_w,G_* represent the mass transfer coefficient of salt in the liquid phase and the mass transfer coefficient of water in the gas phase, respectively. The mass transfer coefficients are calculated according to the relationships reported in Appendix A and Appendix B.

The composition of water vapor at the liquid/membrane interface (*y_w,Lm_*) can be calculated with the modified Raoult’s law (Equation (8)), which takes into account the non-ideality of salt solutions:

(8) $$y_{w,Lm}\;P_{G}=P_{w}^{*}(T_{Lm})\;\gamma_{w,Lm}(T_{Lm},x_{w,Lm})\;x_{w,Lm}$$

in which (*γ_w,Lm_*) represents the activity coefficient of water, which should be calculated at the conditions existing at the liquid/membrane interface.

Finally, in addition to evaporation at the liquid/membrane interface, heat transfer through the liquid feed and gas phase boundary layers must be considered, in addition to heat conduction through the membrane. The net heat flux per unit length for a single capillary (*Q*′*_net_*) transferring through the membrane and the thermal boundary layer of the gas phase is represented by Equation (9a). The heat balance at the liquid/membrane interface is represented by Equation (9b), taking into account liquid evaporation:

(9) $$\begin{array}{cc} Q_{net}^{\prime }=k_{m}^{cond.}\left(T_{Lm}-T_{Gm}\right)\pi d_{lm,m}=h_{G}\left(T_{Gm}-T_{Gb}\right)\pi d_{OUT} & a)\\ Q_{L}^{\prime }=h_{L}\left(T_{Lb}-T_{Lm}\right)\pi d_{IN}=Q_{net}^{\prime }+N_{w}^{\prime }\;\lambda_{w}(T_{Lm}) & b) \end{array}$$

where *h_L_* and *h_G_* represent the convective heat transfer coefficients of the liquid side and of the gas side, respectively. *λ_w_*(*T_Lm_*) is the molar latent heat of vaporization of water that must be calculated at the temperature existing at the liquid/membrane interface.

($k_{m}^{cond.}$) represents a sort of pseudo-thermal conductivity of the membrane that should be calculated accounting for the porosity and thickness of each layer, for the thermal conductivity of the air inside the pores ($k_{G}^{cond.}$) and for the thermal conductivity of the solid portion ($k_{solid}^{cond.}$).

Using the morphological parameters of each layer obtained from the LBL characterization, as given in Table 2, $k_{m}^{cond.}$ should be expressed by the relations reported in Equation (10):

(10) $$\frac{1}{k_{m}^{cond.}\;d_{lm,m}}=\sum_{j=1}^{3,s}\frac{1}{k_{j}^{cond.}\;d_{lm,j}};k_{j}^{cond.}\cdot \delta_{j}=\left(1-\varepsilon_{j}\right)k_{solid}^{cond.}+\varepsilon_{j}\;k_{G}^{cond.}$$

where “*j*” represents a single layer, analogous to what was done in Equation (4).

It is important to note that Equations (9) and (10) are rather general. However, in the present case, some simplifications can be made. Since air thermal conductivity (~0.02 Wm^−1^K^−1^) is very negligible, compared to the conductivity of solid titania (~7.8 Wm^−1^K^−1^) (Appendix B), heat conduction across the membrane is controlled by conductivity of the solid. Because of the very thin layer of the membrane, $k_{m}^{cond.}$ can be estimated to be in the range from 1700 to 2400 Wm^−2^K^−1^; this value, compared with the heat transfer coefficient values in the gas phase, which are typically in the range from 1 to 10 Wm^−2^K^−1^, allows the heat conduction across the membrane to be neglected with respect to convective transfer in the sweeping gas. Consequently, Equation (10) can be neglected, the membrane temperature (*T_m_*) can be calculated as (*T_m_ = T_Lm_*) and Equation (9) can be simplified into Equation (11).

(11) $$h_{L}\left(T_{Lb}-T_{Lm}\right)\pi d_{IN}=N_{w}^{\prime }\;\lambda_{w}(T_{Lm})+h_{G}\left(T_{Lm}-T_{Gb}\right)\pi d_{OUT}$$

With this conclusion, there is no need to distinguish between the use of morphological parameters calculated by the LBL method or by the AMM method.

It is important to note that the model presented here does not contain any adjustable parameter. In fact, the membrane parameters required to calculate the mass transfer coefficient of the membrane (*k_w,m_*) are obtained from gas permeation measurements, independent of SGMD operations. Indeed, the same parameters can be used to simulate and/or describe any MD process, e.g., both direct contact and vacuum membrane distillation operations.

### 3.2. Module Simulation

Since the bundles are in a shell and tube configuration without baffles, each module is simulated assuming a plug flow model both for the liquid and gas streams, according to parallel flow occurring along the effective module length (*L_eff_*) (Figure 1c) between the inlet and outlet nozzles of the shell.

Considering the counter-current configuration, the coordinate system is chosen so that the sweeping gas flows in the positive axial direction, while the liquid flows in the opposite direction, as illustrated by the diagram in Figure 1d. The mass, heat and momentum balance equations with the corresponding boundary conditions are given in Table 5 and Table 6. Some auxiliary variables, such as the liquid velocity in the lumen side (*v_L_*), the interstitial gas velocity (*v*_0,*G*_), and the friction factor (*f*) are also defined.

It is worth noting that Equation (22) is derived from the momentum balance in the shell side in the case of laminar flow according to the equivalent annulus theorem introduced in [35], which is valid, provided that the fibers are uniformly and not tightly packed, as in the present case.

The system of equations given in Table 5 and Table 6 is supplemented using the local model equations (Equations (3)–(11)), under the respective assumptions. The correlations for calculating the transport coefficients for parallel flow are summarized in Appendix A.

The numerical solution of the equations was performed by discretizing the module axially (in the z-direction) for both liquid and gas streams, assuming uniform hydrodynamics in the permeate side. A dedicated MATLAB code was written specifically to perform the computational steps. Similar discretization methods have been used and validated in the literature for SGMD [25,36,37] and other MD configurations [38,39].

The final results are reported as modeled water flux (*J_w_*) values, as defined in Equation (23), referring to the internal area of the module, so as to be comparable with the experimental data, elaborated according to the procedure represented by Equation (1).

(23) $$J_{w}=\frac{M_{w}}{\pi d_{IN}L_{eff}}\int_{0}^{L_{eff}}N_{w}^{\prime }(z)dz$$

## 4. Results and Discussion

**Figure 4:**
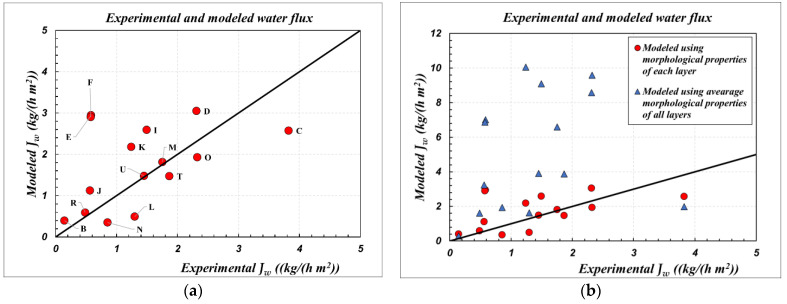
Parity chart for comparison of experimental SGMD results, corresponding to the conditions reported in Table 4, with the simulations obtained by using membrane parameters from: (**a**) LBL method (Table 2); (**b**) LBL method (Table 2); and AMM method (Table 3).

This section reports the results of simulations performed with the model presented in Section 3. A comparison between calculated flux values (modeled *J_w_*) for the same test conditions given in Table 4 and the corresponding experimental flux values (experimental *J_w_*) is first reported. Simulations were performed with the membrane parameters obtained according to the LBL and AMM method. The results are shown in Figure 4.

An interesting agreement between the fluxes modeled with the parameters obtained by the LBL method and the corresponding experimental values can be seen, whereas modeling with the average membrane parameters leads to a strong overestimation of the flux. For most of the cases analyzed, the discrepancy between the fluxes modeled with the LBL parameters and the experimental ones (Figure 4a) is in the range +/−30%. In contrast, the water flux is overestimated by a factor of two to eight when modeled with the AMM parameters.

Based on these results, two main conclusions can be drawn. First, the set of modeling equations, both for the local membrane transport model and the module simulations, can be considered valid and accepted for the simulation of the SGMD process with aqueous solutions, over a wide temperature range.

Second, it is reiterated, without a shadow of a doubt, that the calculation of membrane parameters according to the usual AMM procedure, as suggested and traditionally performed by [4,17,24], is heavily inadequate. A correct representation of the performance of multilayer ceramic membranes is provided exclusively by morphological characterization, according to the LBL method performed by [23,27].

**Figure 5:**
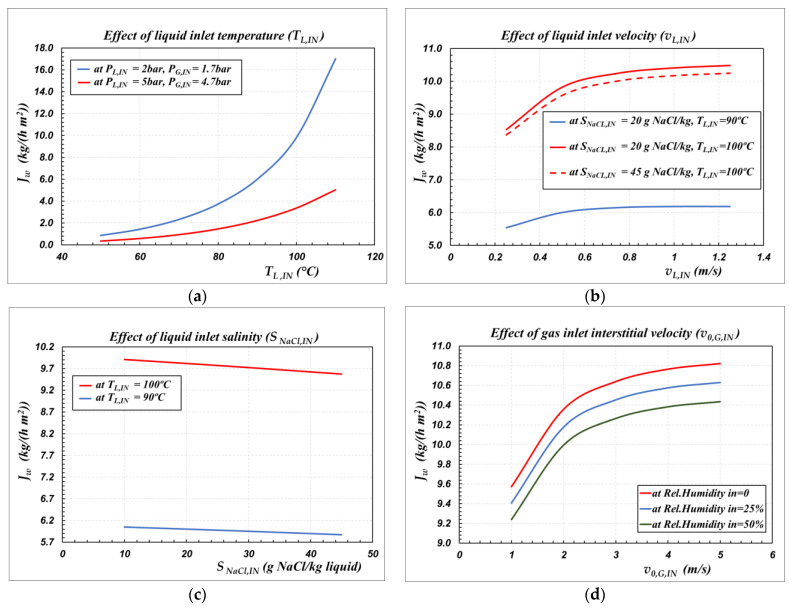
The effect of the inlet operating conditions on the modeled flux in case of countercurrent flow for the bundle B2758 at the operating conditions reported in Table 7. (**a**) Liquid inlet temperature; (**b**) Liquid inlet velocity; (**c**) Liquid inlet salinity; (**d**) Gas inlet interstitial velocity.

Finally, given the good quality of the model equations, a simulation was performed to study the effect of operating conditions on water flux in SGMD for a reference case. The example is that of SGMD of NaCl-water solutions through the B2758 bundle, operated in countercurrent flow, with the liquid in the lumen side and the air in the gas side. The study was conducted by varying temperature, pressure, salinity, and velocity of the liquid stream and by varying temperature, pressure, velocity, and relative humidity of the gas stream. The results are reported in Figure 5, and the corresponding legend is reported in Table 7.

The effects of liquid temperature and of gas pressure are illustrated in Figure 5a. At a given sweeping gas pressure, the flux increases exponentially with the temperature of the inlet liquid, whereas it decreases as the sweeping gas pressure increases, at a given liquid temperature. This is a typical behavior, since the water flux is directly related to the partial pressure difference of water across the membrane, which, in turn, depends on the vapor pressure of water at the feed/membrane interface and on the total pressure value in the gas phase. Indeed, re-arranging Equation (3) and accounting of the liquid-vapor equilibrium at the liquid/membrane interface (Equation (8)), the molar flux of water per unit length of a single capillary (*Nʹ_w_*) can also be expressed by the relationships reported in Equation (24):

(24) $$\begin{array}{l} N_{w}^{\prime }=\frac{k_{w,m}\;P_{G}}{R_{g}T_{m}}\ln\left(\frac{P_{G}-y_{w,Gm}P_{G}}{P_{G}-y_{w,Lm}P_{G}}\right)\pi d_{lm,m}=\frac{k_{w,m}\;P_{G}}{R_{g}T_{m}}\times \frac{y_{w,Lm}P_{G}-y_{w,Gm}P_{G}}{\Delta P_{lm,a}}\pi d_{lm,m}\\ \Delta P_{lm,a}=\frac{\left(P_{G}-y_{w,Gm}P_{G})-(P_{G}-y_{w,Lm}P_{G}\right)}{\ln\frac{P_{G}-y_{w,Gm}P_{G}}{P_{G}-y_{w,Lm}P_{G}}};\;y_{w,Lm}\;P_{G}=P_{w}^{*}(T_{Lm})\;\gamma_{w,Lm}(T_{Lm},x_{w,Lm})\;x_{w,Lm} \end{array}$$

in which *ΔP_lm,a_* represents the logarithmic mean partial pressure of air across the membrane, which is frequently incorporated into the mass transfer coefficient [4].

The effect of liquid velocity within the lumen side on the water flux is reported in Figure 5b. Apparently, increasing the liquid velocity typically means to decrease the heat and mass transfer resistances in the liquid side, and it results in improved water flux: it is interesting to observe that the effect is important at velocities lower than 0.6 m/s, whereas relatively modest increases of fluxes are obtained operating at velocities higher than 0.6 m/s. Thus, an optimal operating range of velocity in the liquid is inferred to be in the range of 0.6 to 0.8 m/s.

The effect of feed salinity is reported in Figure 5c, in which a wide range of salinity is represented, which varies from concentrated brackish waters to very salted sea waters. Obviously, at constant temperature, as the salinity increases, the water activity (*γ_w,Lm_ x_w,Lm_*) of Equation (24) decreases and the water flux decreases. However, the effect is not remarkable in the salinity range investigated; the effect of increasing temperature from 90 to 100 ºC can far outweigh the effect of increasing salinity from 10 to 45 g/kg.

Finally, Figure 5d puts in evidence the additional role of the velocity of the sweeping gas on flux. Moreover, in this case, increasing the gas velocity typically means to decrease the heat and mass transfer resistances in the gas side, and it results in improved water flux. The effect is important at velocities lower than 3 m/s, whereas lower increases of fluxes are obtained operating at velocities higher than 3 m/s. Therefore, an optimal operating range of velocity in the gas is inferred to be in the range of 2 to 3 m/s.

The negative effect of the water vapor partial pressure in the permeate side can also be shown in Figure 5d, where, under the same operating conditions, a decrease in relative humidity in the inlet sweeping gas stream would increase the obtained flux.

From this preliminary study, it can be seen that four process optimization parameters control the process with multilayer ceramic membranes: the liquid temperature and velocity and the gas pressure and velocity. It should be noted that the liquid temperature also affects the gas phase operating pressure, since non-wetting of the membrane must be ensured. Increasing the temperature of the liquid results in a decrease in *LEP_min_*, and, therefore, to prevent wettability of the membrane, the P_L_-P_G_ difference must be decreased, which implies an increase in pressure in the gas. Since the flux decreases with increasing gas pressure, while it increases with increasing liquid temperature, it is clear that a functional optimum will exist between liquid temperature and gas pressure. A detailed analysis of the process will be needed to identify the best operating ranges.

As a final comment, we can observe that the trends depicted in Figure 5 are in relative agreement with the behaviors obtained in experimental and modeling studies in the literature [25,36,37,40,41,42,43]. Regarding the application of SGMD with inorganic membranes, minimal experimental data obtained with prototype membranes are available. However, some results are comparable with the data shown in Figure 5, despite considerable variability in the values. For example, in the SGMD of 40 g/kg NaCl-water solutions with dry nitrogen, the water flux ranged from 11 kg/(m^2^ h) with α-Si_3_N_4_ membranes grafted with dimethyl-dichlorosilane [41] up to 21 kg/(m^2^ h) with alumina membranes grafted with 1H, 1H, 2H, 2H-perfluorooctyltriethoxysilane [43].

## 5. Conclusions

Experimental and modeling studies of SGMD with salt-water solutions using multilayer ceramic bundles were conducted with the aim of identifying the most appropriate morphological characterization method.

Typical equations describing heat and mass transfer in SGMD of salt-water solutions were introduced and adapted to the case of a multilayer membrane made of titania hydrophobized with FAS to simulate the performances of capillary bundles. The description of mass transfer across the membrane was performed by using different values of morphological membrane parameters, calculated from the elaboration of gas permeance data according to the layer-by-layer (LBL) and the average-membrane-morphology (AMM) methods.

Comparing the model simulations with the experimental results, the model values estimated by the LBL method better agree with the experimental results. This indicates that using the average properties of all membrane layers for the studied bundles, as it is generally suggested by other authors, would be considered a very rough assumption that could lead to inaccurate flux estimates. The LBL method is much more appropriate for these membranes, mainly because the membrane layers possess completely different ranges of morphological properties.

The model results also show that the flux depends on the concomitant effects of temperature and velocity of the liquid phase and of pressure and velocity of the gas phase. The liquid velocity should be kept above 0.6 m/s, while the gas velocity should be kept in the range of 2 to 3 m/s. The opposite effect of the liquid temperature and of the gas phase pressure in determining the water flux clearly indicates that a functional optimum will exist between these variables.

## Figures and Tables

**Table 1:** Geometric parameters and *LEP_min_* values at the corresponding temperature of the capillary bundles.

Code	*d_IN_* (mm)	*d_OUT_* (mm)	*N_f_* (fibers)	*L_tot_* (cm)	*d_S_* (cm)	*L_eff_* (cm)	*A_IN_* (cm^2^)	*LEP_min_* (at T) (bar)
B2754	1.56	3.20	37	20	3.60	13	363	4.2 (25 °C)
B2755	1.56	3.20	37	20	3.60	13	363	4 (25 °C)
B2756	1.56	3.20	37	20	3.60	13	363	6.2 (25 °C)
B2888	1.9	3.54	37	20	3.60	13	442	0.30–0.39 (130 °C) ^§^
B2758	1.9	3.20	22	20	2.50	17	263	6.9 (25 °C) ^§^

^§^ from [21].

**Table 2:** Morphological properties of the four layers of the capillary bundles, as estimated by the LBL method [23].

	Layer 3			Layer 2			Layer 1			Support		
Code	*d_p_* (nm)	(*ε*/*τ*)	*δ* (µm)	*d_p_* (nm)	(*ε*/*τ*)	*δ* (µm)	*d_p_* (nm)	(*ε*/*τ*)	*δ* (µm)	*d_p_* (nm)	(*ε*/*τ*)	*δ* (µm)
B2754	548	0.0029	10	250	0.34	30	800	0.20	30	4500	0.11	750
B2755	534	0.0032	10	250	0.34	30	800	0.20	30	4500	0.11	750
B2756	435	0.0044	10	250	0.34	30	800	0.20	30	4500	0.11	750
B2888	328	0.0069	10	250	0.34	30	800	0.20	30	4500	0.11	750
B2758	68	0.084	10	250	0.34	30	800	0.20	30	4500	0.11	580

**Table 3:** Average membrane morphological properties of the capillary bundles, as estimated by the AMM method [23].

	Average Values	
Code	*dp_m_* (nm)	(*ε*/*τ*)
B2754	468	0.27
B2755	1232	0.053
B2756	354	0.44
B2888	337	0.38
B2758	87	3.414

**Table 4:** Operating conditions in SGMD of NaCl-water solutions (symbols refer to Figure 2 and Notation).

	Liquid Inlet to Tube-Side (L_in_)						Gas Inlet to Shell-Side (Gin)				
Trial	T2 (°C)	P3 (bar)	F3 (L/h)	S_NaCl_ (g/kg)	v_L,IN_ (m/s)	ΔP (mbar)	T1 (°C)	P1 (bar)	F0 (m^3^_STP_/h)	v_0,G,IN_ (m/s)	Bundle
B	61.5	4.95	100	18.79	0.39	-	43.0	4.10	5.15	0.56	B2755
C	88.9	2.55	100	18.92	0.39	-	49.0	2.20	2.91	0.60	B2755
D	90.9	2.60	100	19.68	0.39	-	61.0	2.25	2.70	0.57	B2756
E	89.9	2.45	100	18.24	0.39	-	51.5	1.90	1.87	0.45	B2754
F	89.6	2.30	100	18.31	0.39	-	55.5	1.90	1.82	0.44	B2754
H	64.6	3.34	100	19.50	0.45	170	41.7	4.05	1.71	0.58	B2758
I	89.7	3.98	100	19.67	0.45	250	56.1	3.95	0.24	0.63	B2758
J	64.1	2.90	100	19.82	0.45	200	43.1	2.70	1.51	0.58	B2758
K	89.5	4.84	100	20.03	0.45	212	60.8	4.86	4.12	0.90	B2758
L	40.9	2.30	100	18.58	0.45	310	39.3	2.13	2.05	0.98	B2758
M	72.6	2.98	100	18.74	0.45	310	52.5	2.88	2.73	1.01	B2758
N	50.3	5.13	104	18.90	0.46	325	44.5	5.00	4.64	0.96	B2758
O	87.1	5.08	105	19.13	0.47	308	64.5	5.10	4.66	1.00	B2758
P	110.3	5.33	103	19.58	0.46	329	69.8	5.23	4.87	1.03	B2758
Q	110.2	5.25	100	19.93	0.45	290	69.3	5.10	4.76	1.03	B2758
R	70.2	5.18	150	17.97	0.40	331	57.3	5.20	4.84	0.43	B2888
S	89.3	5.13	150	18.75	0.40	296	61.5	5.25	4.87	0.44	B2888
T	90.5	5.03	150	19.58	0.40	251	61.8	5.00	4.63	0.44	B2888
U	91.10	5.05	150	19.95	0.40	264	62.0	5.08	4.66	0.43	B2888

**Table 5:** Balance equations in the liquid side (plug flow model) and auxiliary variables (see Figure 1d and Notation).

Equation		Equation
$\frac{d{\dot{n}}_{L}}{dz}=N_{w}^{\prime }\cdot N_{f}\;$	Total mass balance	(12)
$\frac{d{\dot{n}}_{s}}{dz}=0$	NaCl mass balance	(13)
$\frac{dT_{Lb}}{dz}=\frac{Q_{L}^{\prime }\cdot N_{f}}{{\dot{n}}_{L}\;{\tilde{C}}_{p_{,L}}}$	Heat balance	(14)
$\frac{dv_{L}}{dz}=\frac{4N_{w}^{\prime }M_{w}}{\pi d_{IN}^{2}\rho_{L}}$	Liquid velocity	(15)
$\frac{dP_{L}}{dz}=4f\rho_{L}\frac{v_{L}^{2}}{2d_{IN}}\;$ at Re<2300 f=16/Re, at 2300<Re<5000 f=0.079Re−0.25	Pressure drop	(16)
$\begin{array}{c} at\;z=L_{tot}\\ {\dot{n}}_{L}={\dot{n}}_{L,IN},\;{\dot{n}}_{s}=x_{s,Lb,IN}\;{\dot{n}}_{L,IN},T_{Lb}=T_{L,IN},\;P_{L}=P_{L,IN},v_{L}=v_{L,IN} \end{array}$	Boundary conditions	

**Table 6:** Balance equations in the gas side (plug flow model) and auxiliary variables (see Figure 1d and Notation).

Equation		Equation
$\frac{d{\dot{n}}_{G}}{dz}=N_{w}^{\prime }\cdot N_{f}$	Total mass balance	(17)
$\frac{d{\dot{n}}_{a}}{dz}=0$	Air mass balance	(18)
$\frac{d\;T_{Gb}}{dz}=\frac{Q_{net}^{\prime }\cdot N_{f}}{{\dot{n}}_{G}\;{\tilde{C}}_{P,G}}$	Heat balance	(19)
$\left(\frac{1}{\rho_{G}}\right)\frac{d\rho_{G}}{dz}=\left(\frac{-1}{T_{Gb}}\right)\frac{dT_{Gb}}{dz}+\left(\frac{1}{P_{G}}\right)\frac{dP_{G}}{dz}+\left(\frac{M_{w}-M_{a}}{M_{G}}\right)\frac{dy_{w,Gb}}{dz}$	Ideal gas law	(20)
$\rho_{G}\left(\frac{dv_{0,G}}{dz}\right)+v_{0,G}\left(\frac{d\rho_{G}}{dz}\right)=\frac{4N_{w}^{\prime }M_{w}}{\pi \left(\frac{d_{S}^{2}}{N_{f}}-d_{OUT}^{2}\right)}$ $v_{0,G}=\frac{{\dot{n}}_{G}M_{G}/\rho_{G}}{\left(1-\varepsilon_{p}\right)\left(\frac{\pi }{4}d_{S}^{2}\right)},\varepsilon_{p}=N_{f,tot}{\left(\frac{d_{OUT}}{d_{S}}\right)}^{2}$	Gas phase velocity	(21)
$\frac{dP_{G}}{dz}=\frac{8N_{f}\eta_{G}v_{0,G}}{d_{S}^{2}\left(\frac{1}{2}\left(\frac{\ln\left(\varepsilon_{p}\right)}{1-\varepsilon_{p}}+\frac{3-\varepsilon_{p}}{2}\right)\right)}\;$	Pressure drops (equivalent annulus model [35]	(22)
$\begin{array}{c} at\;z=0\\ {\dot{n}}_{G}={\dot{n}}_{G,IN},\;{\dot{n}}_{a}={\dot{n}}_{a,IN},\;T_{Gb}=T_{G,IN},\;P_{G}=P_{G,IN}\\ v_{0,G}=v_{0,G,IN},\;\rho_{G}=\rho_{G,IN},\;y_{w}{}_{,Gb}=y_{w,Gb,IN} \end{array}$	Boundary conditions	

**Table 7:** Operating conditions used for simulations reported in Figure 5a–d.

Figure	5a	5b	5c	5d
*T_L,IN_* (°C)	*	*	*	100
*P_L,IN_* (bar)	*	2	2	2
*S_NaCl,IN_* (g/kg)	20	*	*	45
*v_L,IN_* (m/s)	0.5	*	0.5	0.5
*T_G,IN_* (°C)	45	45	45	45
*P_G,IN_* (bar)	*	1.7	1.7	1.7
Relative humidity of air (%)	0	0	0	*
*v_0,G,IN_* (m/s)	1	1	1	*

* Variable condition indicated in the corresponding figure.

## Data Availability

The data supporting the findings of this study are available upon reasonable request.

## List of Symbols

**Latin Letters**		**SI Units**
$A_{IN}$	Inner surface Area	[m^2^]
${\tilde{C}}_{p},{\hat{C}}_{p}$	Molar, mass, heat capacity at constant pressure	[J mol^−1^ K^−1^], [J kg^−1^ K^−1^]
$d_{IN}$	Inner diameter of a fiber	[m]
$d_{OUT}$	Outer diameter of a fiber	[m]
$d_{lm,m}$	Logarithmic mean diameter of the membrane $d_{lm,m}=\frac{d_{OUT}-d_{IN}}{\ln\frac{d_{OUT}}{d_{IN}}}$	[m]
$d_{lm,j}$	Logarithmic mean diameter of the membrane layer j	[m]
$d_{S}$	Shell diameter	[m]
$d_{p,j}$	Pore diameter of the membrane layer j	[m]
$d_{pm}$	Mean pore diameter of the membrane	[m]
$D_{W,Kn}$	Knudsen diffusion coefficient of water	[m^2^ s^−1^]
$D_{WG}$	Molecular diffusion coefficient of water in gas	[m^2^ s^−1^]
$D_{Weq}$	Equivalent diffusion coefficient of water	[m^2^ s^−1^]
*D*	Molecular diffusion coefficient	[m^2^ s^−1^]
*f*	Fanning factor	[dimensionless]
$G_{in}$	Inlet stream of gas	
$G_{out}$	Outlet stream of gas	
$Gz_{H},Gz_{M}$	Graetz number for heat, mass transfer	[dimensionless]
*h*	Convective heat transfer coefficient	[W m^−2^ K^−1^]
$J_{w}$	Mass flux of water across the membrane (defined in Equation (2))	[kg m^−2^ s^−1^)
$k_{w}$	Mass transfer coefficient of water	[m s^−1^]
$k_{S,L}$	Mass transfer coefficient of salt in liquid	[m s^−1^]
$k^{\mathrm{cond}.}$	Thermal conductivity coefficient	[W m^−1^ K^−1^]
$k_{m}^{cond.}$	Pseudo-thermal conductivity of the membrane (defined in Equation (9a))	[W m^−2^ K^−1^]
$L_{\mathrm{eff}}$	Effective length of membrane module (Figure 1c)	[m]
$L_{\mathrm{tot}}$	Total length of membrane module	[m]
$L_{\mathrm{in}}$	Inlet stream of liquid	
$L_{\mathrm{out}}$	Outlet stream of liquid	
$m_{w,\mathrm{liquid}\;\mathrm{phase}}$	Mass of water in the liquid side	[kg]
$m_{\mathrm{tot},\mathrm{liquid}\;\mathrm{phase}}$	Total mass of solution in the liquid side	[kg]
*M*	Molar mass	[kg mol^−1^]
$\overset{\cdot }{n}$	Molar flow rate	[mol s^−1^]
$N_{w}^{\prime }$	Transmembrane Molar flow rate of water per unit length per fiber	[mol m^−1^ s^−1^]
$N_{f}$	Number of fibers	[dimensionless]
*Nu*	Nusselt number	[dimensionless]
*P*	Pressure	[Pa]
*Pr*	Prandtl number	[dimensionless]
$P_{w}^{*}$	Vapor pressure of water	[Pa]
$Q_{L}^{\prime }$	Heat flow rate per unit length per fiber in the liquid thermal boundary layer	[W m^−1^]
$Q_{\mathrm{net}}^{\prime }$	Net transmembrane heat flow rate per unit length per fiber	[W m^−1^]
*Re*	Reynolds number	[dimensionless]
$R_{g}$	Universal gas constant	[J mol^−1^ K^−1^]
$S_{\mathrm{Nacl}}$	Salinity of NaCl solution	[g NaCl kg^−1^ Solution]
*Sc*	Schmidt number	[dimensionless]
*Sh*	Sherwood number	[dimensionless]
*T*	Temperature	[K]
$v_{L}$	Liquid velocity in lumen-side (defined in Equation (15))	[m s^−1^]
$V_{0,G}$	Gas interstitial velocity in shell-side (defined in Equation (21))	[m s^−1^]
*x*	Mole fraction in liquid phase	[dimensionless]
*y*	Mole fraction in gas phase	[dimensionless]
*z*	Axial coordinate in membrane module	[m]
**Greek Letters**		**SI Units**
$\gamma_{w,Lm}$	Activity coefficient of water at liquid/membrane interface	[dimensionless]
δ	Thickness	[m]
${\left(\varepsilon /\tau \right)}_{j}$	Porosity-tortuosity ratio of the membrane layer j	[dimensionless]
${\left(\varepsilon /\tau \right)}_{m}$	Mean porosity-tortuosity ratio of the membrane	[dimensionless]
$\varepsilon_{p}$	Packing factor of the membrane module	[dimensionless]
η	Dynamic viscosity	[Pa s]
λ	Molar latent heat of vaporization	[J mol^−1^]
ρ	Density	[kg m^−3^]
**Superscripts and Subscripts**		
*a*	Air	
*G*	Gas side	
*Gb*	At gas bulk	
*Gm*	At gas/membrane interface	
*IN*	Inlet section	
*j*	Layer j (j = S for support, j = 1 for layer1, j = 2 for layer 2, j = 3 for layer 3)	
*L*	Liquid side	
*Lb*	At liquid bulk	
*Lm*	At liquid/membrane interface	
*s*	Salt	
*solid*	Solid portion of the membrane	
*w*	Water	

## Appendix A. Mass and Heat Transfer Correlations

Table A1 shows the correlations used for the calculation of heat and mass transfer coefficients of model equations reported in Section 3. Chilton–Colburn analogy between heat and mass transfer was applied to estimate the Sherwood number; citation numbers refer to the corresponding bibliography for heat transfer. The definition of the dimensionless number is reported in Table A2.

**Table A1:** Correlations for heat and mass transfer in forced convection in unbaffled shell and tube configuration.

Side	Correlation	Validity Range	Reference
Tube	$\begin{array}{c} Nu=3.66+\frac{0.0668\;Gz_{H}}{1+0.04\;Gz_{H}^{2/3}}\\ Sh=3.66+\frac{0.0668\;Gz_{M}}{1+0.04\;Gz_{M}^{2/3}} \end{array}$ (A.1)	$\begin{array}{l} Re<2100\\ Gz_{H},Gz_{M}<100 \end{array}$	[44,45]
	$\begin{array}{l} Nu=0.116\left(Re_{}^{2/3}-125\right)Pr_{}^{1/3}\left[1+{\left(\frac{d_{IN}}{L_{eff}}\right)}^{2/3}\right]\\ Sh=0.116\left(Re_{}^{2/3}-125\right)Sc_{}^{1/3}\left[1+{\left(\frac{d_{IN}}{L_{eff}}\right)}^{2/3}\right] \end{array}$ (A.2)	$\begin{array}{l} 2100<Re<10^{4}\\ 60<\frac{L_{tube}}{d_{IN}}<250 \end{array}$	[46,47]
Shell (parallel flow)	$\begin{array}{l} Nu=0.128d_{eq}^{0.6}Re_{}^{0.6}Pr_{}^{1/3}\\ Sh=0.128d_{eq}^{0.6}Re_{}^{0.6}Sc_{}^{1/3} \end{array}$ (A.3) $d_{eq}=\frac{\left(d_{S}^{2}-N_{f}d_{OUT}^{2}\right)}{\left(d_{S}+N_{f}d_{OUT}\right)}$(in inches)	$\begin{array}{l} 80<d_{eq}Re<2\times 10^{4}\\ inches \end{array}$	[44]

**Table A2:** Dimensionless numbers used in Table A1.

Heat Transfer	Mass Transfer
$Re=\frac{\rho \;v^{*}\;l^{*}}{\eta }$	
$Nu=\frac{h\;l^{*}}{k^{cond.}}$	$Sh=\frac{k\;l^{*}}{D_{}}$
$Pr=\frac{\eta \;{\hat{C}}_{p}}{k^{cond.}}$	$Sc=\frac{\eta \;}{\rho \;D_{}}$
$G\;z_{\;H}=Re\;Pr\frac{d_{IN}}{L_{eff}}$	$G\;z_{\;M}=Re\;Sc\frac{d_{IN}}{L_{eff}}$
$l^{*}=d_{IN}\;(for\;tube),\;l^{*}=d_{OUT}\;(for\;shell),\;v^{*}=v_{L}\;(for\;tube),\;v^{*}=v_{0,G}\;(for\;shell)$	

## Appendix B. Relevant Chemical-Physical Properties

- Chemical-physical properties of NaCl-water solutions were taken from [48,49];
- Activity coefficient of water in NaCl-water solutions was obtained by the osmotic pressure data reported in [50];
- Chemical-physical properties of gas phase were taken from [45,49];
- Thermal conductivity of titania (the solid portion of the membrane) was taken from [51].
